# Biological N_2_O Fixation in the Eastern South Pacific Ocean and Marine Cyanobacterial Cultures

**DOI:** 10.1371/journal.pone.0063956

**Published:** 2013-05-23

**Authors:** Laura Farías, Juan Faúndez, Camila Fernández, Marcela Cornejo, Sandra Sanhueza, Cristina Carrasco

**Affiliations:** 1 Laboratory of Oceanographic and Climate Processes (PROFC), Department of Oceanography, University of Concepcion, and Center for Climate Change and Resilience Research (CR2), Concepción, Chile; 2 Graduate Program in Oceanography, Department of Oceanography, University of Concepcion, Concepcion, Chile; 3 UPMC Univ Paris 06 and CNRS, UMR 7621, LOMIC, Observatoire Océanologique, Banyuls s/mer, France; Uppsala University, Sweden

## Abstract

Despite the importance of nitrous oxide (N_2_O) in the global radiative balance and atmospheric ozone chemistry, its sources and sinks within the Earth’s system are still poorly understood. In the ocean, N_2_O is produced by microbiological processes such as nitrification and partial denitrification, which account for about a third of global emissions. Conversely, complete denitrification (the dissimilative reduction of N_2_O to N_2_) under suboxic/anoxic conditions is the only known pathway accountable for N_2_O consumption in the ocean. In this work, it is demonstrated that the biological assimilation of N_2_O could be a significant pathway capable of directly transforming this gas into particulate organic nitrogen (PON). N_2_O is shown to be biologically fixed within the subtropical and tropical waters of the eastern South Pacific Ocean, under a wide range of oceanographic conditions and at rates ranging from 2 pmol N L^−1^ d^−^ to 14.8 nmol N L^−1^ d^−1^ (mean ± SE of 0.522±1.06 nmol N L^−1^ d^−1^, n = 93). Additional assays revealed that cultured cyanobacterial strains of *Trichodesmium* (H-9 and IMS 101), and *Crocosphaera* (W-8501) have the capacity to directly fix N_2_O under laboratory conditions; suggesting that marine photoautotrophic diazotrophs could be using N_2_O as a substrate. This metabolic capacity however was absent in *Synechococcus* (RCC 1029). The findings presented here indicate that assimilative N_2_O fixation takes place under extreme environmental conditions (i.e., light, nutrient, oxygen) where both autotrophic (including cyanobacteria) and heterotrophic microbes appear to be involved. This process could provide a globally significant sink for atmospheric N_2_O which in turn affects the oceanic N_2_O inventory and may also represent a yet unexplored global oceanic source of fixed N.

## Introduction

Nitrous oxide (N_2_O) is an important greenhouse gas that contributes to stratospheric ozone depletion. The transfer of this gas within the Earth System is intimately linked to physical and biological processes occurring in the ocean, atmosphere and soils. In the ocean, N_2_O saturation levels depend upon oceanographic variables such as temperature and salinity while processes producing N_2_O are mainly being controlled by organic matter and dissolved O_2_ [1]. A detailed understanding of its biological production and consumption is necessary to predict the effects of long term changes in N_2_O on the Earth’s climate [2].

According to current knowledge, nitrification performed by bacteria [3] and archaea [4], via aerobic NH_4_^+^ oxidation or nitrifier denitrification (the pathway in which NH_4_^+^ is oxidized to NO_2_^−^ followed by the reduction of NO_2_^−^ to NO, N_2_O, and N_2_ [5]) is the main process responsible for most of the N_2_O production under oxic or even microaerophilic conditions; whereas partial denitrification, via the anaerobic reduction of NO_2_^−^ to N_2_O, can produce this gas under suboxic conditions [3], [6].

Contrary to its production, N_2_O can only be consumed by photolysis in the stratosphere [7] and by canonical denitrification via dissimilative reduction of N_2_O to N_2_, a pathway that only exists under anoxic condition [3], [6], [8]. However, early studies suggest that N_2_O, and even NO_2_^−^, could act as substrates for the enzymatic nitrogenase complex (NifH) [9], [10]. Indeed, it is also known that given the properties of the NifH, certain diazotrophs are able to reduce not only N_2_, but also other multi-bonded substrates, such as acetylene, azide, cyanide, methyl isocyanide and even N_2_O [11], [12]. Regarding N_2_O, this molecule could bind to metals belonging to the NifH complex through the N-bound nitro form and the N-O bond form [13]. However, there is no direct evidence so far that indicates how or where the N-O bond containing molecule N_2_O binds to NifH, or that indicates the chemical mechanism of its reduction.

The eastern South Pacific (ESP) region hosts the most extreme range of biogeochemical conditions in the global ocean, from the very eutrophic, nitrate-rich and oxygen-poor waters of the Peruvian and Chilean coastal upwelling (CU ∼71°–76°W) to oxygenated and severely nutrient-limited waters of the central subtropical Pacific gyre (STG ∼110°W) [14]. Dissolved O_2_ vertical distribution denotes the well-known oxygen minimum zone (OMZ),which has relatively shallow suboxic-anoxic waters (0≥ O_2_≤11 µmol L^−1^) in subsurface waters off Peru and northern Chile [15] and also the oxygenated waters towards the subtropical gyre and south of ∼37°S. N_2_O concentrations in seawater and its concomitant exchange across air-sea interface also reflect the previously mentioned biogeochemical gradients in the ESP. Thus, N_2_O content in the ESP can be as high as 400% saturation with strong effluxes within coastal upwelling and sub-saturated or slightly supersaturated N_2_O levels (∼80–105%) with near zero air-sea fluxes within the subtropical gyre (STG) [16], [17]. A number of studies reports sub-saturated N_2_O values in surface waters throughout the world’s major oceans (Table S1 and Text S1); these low values have been assumed to be analytical artifacts [18] given the absence of any known chemical or biological mechanism able to remove N_2_O from surface waters. In suboxic subsurface waters, in contrast, such as those found in the OMZ of the ESP (at depths from 50 to 400 m), sub-saturated N_2_O concentrations (as low as 40–60%) have been solely attributed to canonical denitrification, which is so far the only known process able to use N_2_O as an electron acceptor instead of dissolved O_2_. Canonical denitrification along with anaerobic NH_4_^+^ oxidation (anammox), both processes particularly active in the ESP [19], [20], are the main sinks for fixed N budgets.

There has been long-standing uncertainty as to whether or not the ocean is losing N faster than it is being incorporated via marine N_2_ fixation [8], [21]. Important advances in the understanding of regulation, rates and the microorganisms involved in N_2_ fixation have been made in recent years [22]–[24]. These studies indicate that NifH is present in a diverse range of microbial groups which include the well-studied diazotrophic cyanobacteria, diatom–diazotroph assemblages and gammaproteobacteria [23], [25], [26]. In fact, diazotrophs display high levels of metabolic diversity, much greater than previously thought, among which can be found not only well-known photoautotrophs but also chemo- and heterotrophic diazotrophs [27]. Recent results showing the expression of nitrogenase gene (*nifH*), suggest that heterotrophic bacteria dominate the diazotrophic community in the oligotrophic waters of the western South Pacific [28] and even in the OMZ of the Arabian Sea [29].

The possible occurrence of biological N_2_O fixation or rather the well-known process which removes N_2_O was investigated using an enriched, labeled substrate (^15^N_2_O). This process was explored using field samples and cultures of marine cyanobacteria such as *Trichodesmium* and *Crocosphaera*. These bacteria, particularly *Trichodesmium*, are present in most tropical and subtropical gyres, being the dominant diazotrophic species and having global significance in relation to the introduction of new N into the ocean [30], [31].

## Results and Discussion

**Figure 1:**
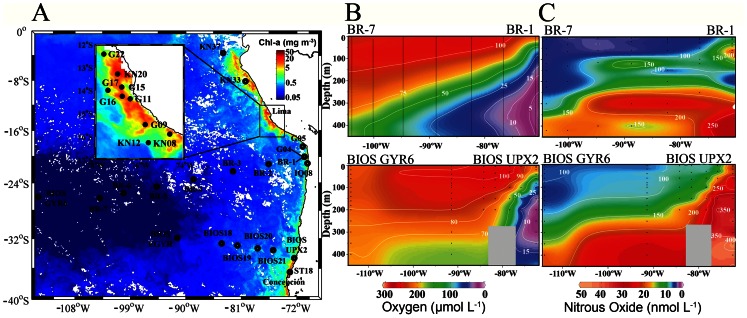
Study area showing: A) The location of the sampling stations superimposed on a background illustrating mean surface Chlorophyll concentrations for the 2005–2011 period (Color data is available at http://oceancolor.gsfc.nasa.gov/cgi/l3), Chl-a concentration expressed in mg Chl-a m^−3^ coded according to the color bar); B) Zonal dissolved oxygen transects and its saturation percentage in two transects, from Easter Island to the coast at 20°S (Iquique), and from Easter Island to the coast at 32°S (Valparaiso); and C) Zonal distribution of dissolved N_2_O and its saturation percentages obtained from the same two transects. Color scales show dissolved O_2_ concentrations in (µmol L^−1^), while the solid lines indicate saturation percentages (%).

N_2_O fixation was explored in field experiments carried out during several cruises covering a wide range of geographical locations (13°–36.5°S; 72°–110°W) and depths (from the surface down to 400 m). The study areas have extreme biogeochemical conditions reflected in surface chlorophyll-a (Chl-a; Fig. 1A), dissolved fixed N (mainly NO_3_^−^) and O_2_. The latter variable varies from the oxygenated waters in the subtropical gyre and south of ∼37°S to suboxic-anoxic waters (0≥ O_2_≤11 µmol L^−1^) off Peru and northern Chile. Oxygen deficient waters are clearly observed (Fig. 1B), delimiting an OMZ that has become one of the shallowest and most intense in the world ocean [19]. Oceanographic conditions of the sampled stations are summarized in Table 1.

**Table 1:** Location, water depth along with some oceanographic and meteorological variables/parameters obtained from the sampled stations.

Station	Date mm/dd/yy		Lat. (°S)	Lon. (°W)	Water depth (m)	SST (°C)	Wind (m s^−1^)	Z_m_ (m)	Surface NO_3_^−^ (µmol L^−1^)	Surface PO_4_^3−^ (µmol L^−1^)	Surface N/P ratio	N_2_O fix assay
BIOS GYR6	11/14/04	−26.06		−113.98	3078	23.2	2.87	189	0.05	0.06	0.83	
BIOS EGYR	11/30/04	−31.90		−91.40	2996	18.3	6.47	180	0.13	0.10	1.3	
BIOS 18	12/02/04	−32.66		−84.20	3760	17.5	6.40	174	3.64	0.36	10	
BIOS 19	12/03/04	−32.94		−81.63	4006	17.13	2.45	128	2.74	0.36	7.6	
BIOS 20	12/04/04	−33.32		−78.36	3830	17.4	3.94	125	0.92	0.30	3	
BIOS 21	12/05/04	−33.58		−75.84	4374	16.8	8.57	83	0.06	0.30	0.20	
BIOS UPX2	12/07/04	−34.65		−72.47	1193	12.8	8.90	40	19.48	1.30	15	
KN08	10/20/05	−15.91		−74.65	1370	15.0	5.9	28	9.6	1.7	5.7	*
KN12	10/22/05	−16.28		−75.61	4100	15.9	4.17	27	10.3	1.4	7.4	*
KN20	10/25/05	−13.3		−76.99	885	15.3	6.98	6	7.4	1.9	3.8	*
KN33	10/30/05	−8.17		−80.33	328	17.4	5.76	27	8.9	1.3	7	*
KN37	11/02/05	−3.6		−83.95	3239	18.5	4.06	25	NA	NA	NA	*
G04 (14.6)	02/17/07	−20.06		−70.75	1480	21.9	NA	17	1.4	0.64	2.19	*
G05 (14.14)	02/18/07	−18.5		−71.03	1203	23.9	4.47	12	4.1	0.08	51.3	*
G09 (14.21)	02/19/07	−15.5		−75.75	3135	21.9	7.5	16	NA	0.45	NA	*
G11 (14.47)	02/21/07	−14.38		−76.42	315	17.6	8.1	30	10.7	0.82	13.0	*
G15 (14.66)	02/22/07	−13.87		−76.8	750	19.5	9.7	16	NA	NA	NA	*
G16 (14.74)	02/23/07	−14.27		76.78	789	20.0	11.5	10	6.1	0.07	87.1	*
G17 (14.86)	02/24/07	−14.01		−77.42	5153	20.9	6.7	19	4.9	0.48	10.21	*
G22 (14.100)	02/27/07	−12.43		−77.6	598	21.2	4.0	14	0.6	0.18	3.33	*
IQ08	09/22/08	−21.07		−70.27	2200	15.6	NA	10	0.07	NA	NA	*
ST 18^a^	2009	−36.51		−73.12	92	12.6	6.8	19	11.7	1.13	9.7	*
BR−1	11/20/11	20. 05		−70.47	1900	19.77	8.88	14	0.33	0.94	0.35	*
BR−2	11/25/11	21.10		−76.34	4695	18.0	NA	57	0.31	0.49	0.61	
BR−3	11/27/11	22.15		−82.20	2572	18.0	NA	47	0,45	0.20	0.8	
BR−4	12/01/11	23.27		−88.46	3941	18.7	NA	62	BLD	0.34	0.08	
BR−5	12/04/11	24.33		−94.43	3402	19.7	NA	69	BLD	0.27	>0.01	
BR−6	12/06/11	25.33		−100.08	3181	20.9	NA	52	BLD	0.22	>0.01	
BR−7	12−09/11	26.14		−103.57	2691	21.9	4.11	41	BLD	0.18	>0.01	*

They include Sea Surface Temperature (SST), wind speed, mixing layer depth (Z_m_), surface concentrations of nitrate and phosphate, surface dissolved N/P ratio.

a COPAS time series station. Values averaged on austral spring-summer period.

* Denotes the stations where assimilative N_2_O fixation assays were performed. BLD: Below limit of Detection; NA: Not Available.

N_2_O fixation was observed in 92% of sampled depths, with rates ranging between 2 pmol N L^−1^ d^−1^ and 14.8 nmol N L^−1^ d^−1^ (mean ± SE of 0.522±1.06 nmol N L^−1^ d^−1^, n = 93). These field samples, which came from different trophic (Fig. 1A) and O_2_ regimes (Fig. 1B), were incubated on board under a light gradient (65% to 4% of surface irradiance) and under dark conditions, and with temperature and dissolved O_2_ levels maintained close to those found under *in situ* conditions (Table S2). N_2_O fixation was blocked in control experiments treated with HgCl_2_ (total experiments n = 15), confirming the biological nature of this process, hereafter referred to as assimilative N_2_O fixation.

**Figure 2:**
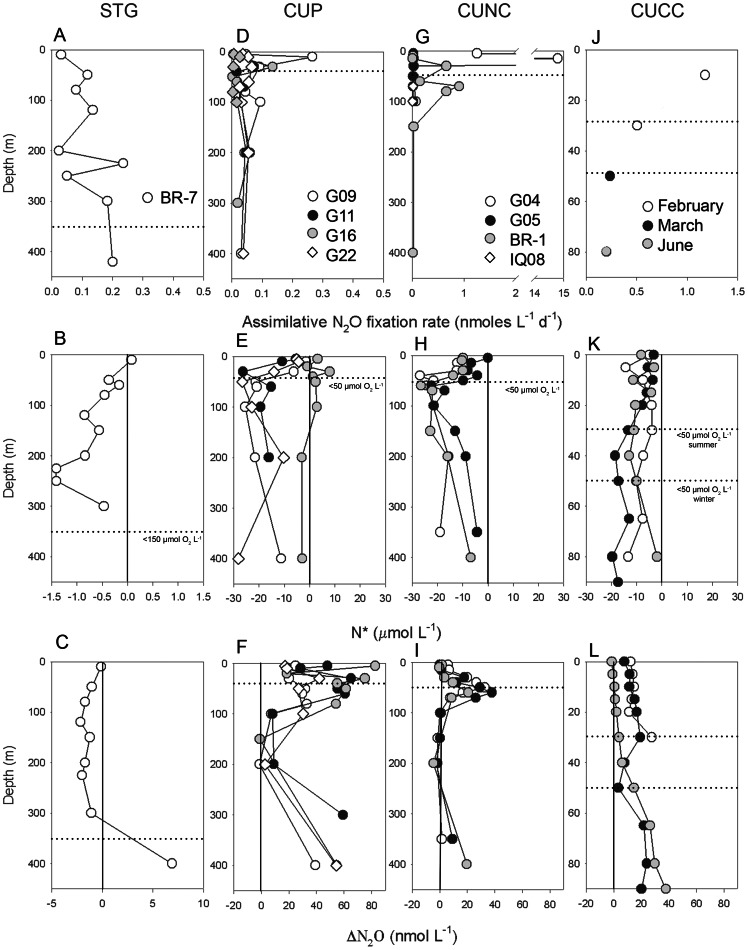
Depth profiles of assimilative N_2_O fixation rates (nmol L^−1^ d^−1^) (A, D, G, J) along with N* (B, E, H, K) and apparent N_2_O production (ΔN_2_O µmol L^−1^) (C, F, I, L). Parameters at selected stations from left to right column are: the STG (St. BR 7); the CUP (Sts. G09, G11, G16 and G22); the CUNC (Sts. G04, G05, IQ08 and BR 1); and the CUCC (St.18 or COPAS sampled in January, February, March, 2009). Note the change in scale in N* and ΔN_2_O for the STG and in the assimilative N_2_O fixation rates between the surveyed locations. Vertical line denotes zero for N* and ΔN_2_O indexes.

Vertical distributions of N_2_O fixation rates are shown in Fig. 2A, D, G, J along with the semi-conservative tracer N* [21] (Fig. 2B, E, H, K) and ΔN_2_O [32] (Fig. 2C, F, I, L). Off Peru (CUP) and northern Chile (CUNC), N_2_O fixation rates peaked at the surface and/or around the base of the oxycline (Fig. 2D, 2G) and were well correlated with fluorescence and particulate organic carbon and nitrogen (POC/PON) distributions (data not shown). Off central Chile (CUCC, Fig. 2J), N_2_O fixation rates were higher in the photic-oxic layer decreasing toward deeper waters (90 m depth). At the STG (BR-7 station, Fig. 2A), remarkably, vertical N_2_O fixation showed a maximum level at the base of the eufothic zone, where chlorophyll fluorescence and POC revealed a maximum level [33]. Additionally, experiments performed in the photic zone during the Big Rapa (BR-1 and BR-7 stations) showed active N_2_O fixation rates under dark as well as *in situ* light conditions (see Table S2), but without any significant differences. This supports the idea that microorganisms assimilating N_2_O are not only photoautotrophs and that part of microbes fixing N_2_O could be heterotrophs.

Table 2 shows ranges of N_2_O fixation rates and their statistics, along with basic biogeochemical variables in predefined areas i.e., the STG and coastal upwelling (CU) centers. There was significant variation in N_2_O fixation rates among the study areas (Kruskal Wallis test, p<0.05), being higher at the CUNC. Further analyses involving a multiple linear regression (p<0.05) showed that 27% of the variation observed in N_2_O fixation rates was linked to dissolved O_2_ concentrations, with higher rates observed at lower O_2_ values; less variance percentage was ascribed to differences in Chl-a and fixed N pools among areas. In this sense, it has been suggested that dissolved O_2_ controls surface diazotrophic activity which could be enhanced in close proximity to water column denitrification areas [34].

**Table 2:** Rates of N_2_O fixation (range and average±SD) along with surface inventories of nitrate (fixed N), nitrous oxide and chlorophyll-a in the sampled area.

	Subtropical South Pacificgyre (STG)	Coastal Upwelling offPeru (CUP)	Coastal Upwelling off northern Chile (CUNC)	Coastal Upwelling off central Chile (CUCC)
Representative for:	10°–30° S	10°–19°S	20°–23°S	35°–37° S
	80°–110°W	71°–76°W	∼71°–72°W	75°–76.5°W
Surface area (km^2^)	6.9×10^6^	5.3×10^5^	1.6×10^3^	1.1×10^4^
Assimilative N_2_O fixation rate(nmol N L^−1^ d^−1^)	0.023–10.64	0.002–0.266	0.002–14.79	0.202–1.172
average±SD	0.825±1.705 (n = 17)	0.051±0.048 (n = 63)	0.485±1.140 (n = 37)	0.530±0.451 (n = 26)
Fixed N inventory(mmol N m^−2^)	2.0–36.5	33.7–150	52.1–120	200–818
N_2_O inventory(µmol N m^−2^)	825–1125	247–1373	500–2061	157–2786*
Chl-a inventory(mg m^−2^)	1.53–20	>500	50–100	250–500
Other features	Deep biome	Partial presence of continental shelf	Non presence of continental shelf	Large continental shelf
	Extremely Fe and N limitation	Moderate Fe and N limitation	Moderate Fe limitation	Non expected Fe limitation Bío-Bío river
	Oxygenated water	Permanent OMZ	Permanent OMZ	Seasonal OMZ

* N_2_O inventories based on data come from the COPAS time series station since 2002 to date.

Assimilative N_2_O fixation rates in the photic layer samples, under different light intensities, averaged 0.493±2.27 nmol N L^−1^ d^−1^ (n = 42). Despite the fact that certain maxima of N_2_O fixation rates are observed in the photic zone, these values were not significantly different (Mann–Whitney test, p = 0.03) from those measured in the aphotic layer, under the occasional influence of the OMZ, which averaged 0.104±0.190 nmol N L^−1^d^−1^ (n = 32).

In addition, all sampled areas in this study share a moderate to severe iron and NO_3_^−^ deficiency [35]–[37] which is reflected in the N* index. Indeed, in each predefined area N* profiles started with values close to zero in surface water and decreased with depth, reaching negative N* values as low as −30 (Fig. 2B, E, H and K). Thus, N* indicates a depletion or deficit of N compared to P, and could be a consequence of the lateral and upward transport of denitrified waters from the OMZ belonging to the ESP, causing a (lower than expected) deviation of the Redfield N:P ratio. If PO_4_^−3^ is forced above the Redfield ratio, N fixing organisms may gain a selective advantage, which increases the inventory of NO_3_^−^. Consequently, the impact of N_2_O fixation may lie in compensating N_2_O loss caused by canonical denitrification. Particularly, the N deficit relative to P is a common pattern observed at the STG [38] (Fig. 2 B) and may be accentuated by weak vertical mixing and very scarce aeolian dust supply toward this region [14]. There, N* values close to zero or negative zero are usually observed within the gyre explaining the predominance of advected denitrification over N-fixation [21], [38].

On the other hand, within the STG (St. BR-7) noticeable negative values of ΔN_2_O in oxygenated water were detected (Fig. 2C). However, these could not be possible by heterotrophic denitrification given the oxygenated condition of the entire water column. Therefore, the ΔN_2_O values suggest that the N_2_O deficit could be caused by assimilative N_2_O consumption. In contrast, in the upwelling of Peru, northern Chile ΔN_2_O values decrease from positive values at surface, peaking sometime at the oxyclines, to close to zero or lightly negative towards the OMZ’s core (Fig. 2F, I); there N_2_O is consumed by canonical denitrification [39].

It is important to note that N_2_ fixation rates were detected at the same stations and depths [40] as the detection of N_2_O fixation rates in our sampled CU areas (in 60% of the samples). In addition, in both CUP and CUNC study areas, dinitrogenase reductase genes *(nifH)* have been found and phylogenetic analysis revealed a diverse diazotrophic community [40] in which *nifH* sequences fell within three of the four known clusters for this gene [41], [42]. Importantly however, no sequences associated with cyanobacteria were found during that study [40] which agrees with nifH transcripts retrieved in the western South Pacific gyre that showed active heterotrophic communities, mainly γ-proteobacteria from cluster I [42], able to fix N_2_ [28].

In order to check whether typical diazotrophic organisms have the same capacity to fix N_2_O as they have for fixing N_2_, three of the most studied strains of marine cyanobacteria [43] belonging to *Trichodesmium* (H-9 and IMS101) and *Crocosphaera* (W-8501) were cultured under laboratory conditions. It is important to note that they used NifH to transform N_2_ into PON, but lacked the *nosZ* genes that encode the catalyzing enzymes of dissimilative N_2_O reduction to N_2_ [44], thereby only leaving assimilative N_2_O fixation as the potential N_2_O consumption process. By contrast, *Synechococcus* (RCC 1029) was used as a negative control, given that this species does not possess either *nifH* or *nosZ* genes in its genome [45].

**Figure 3:**
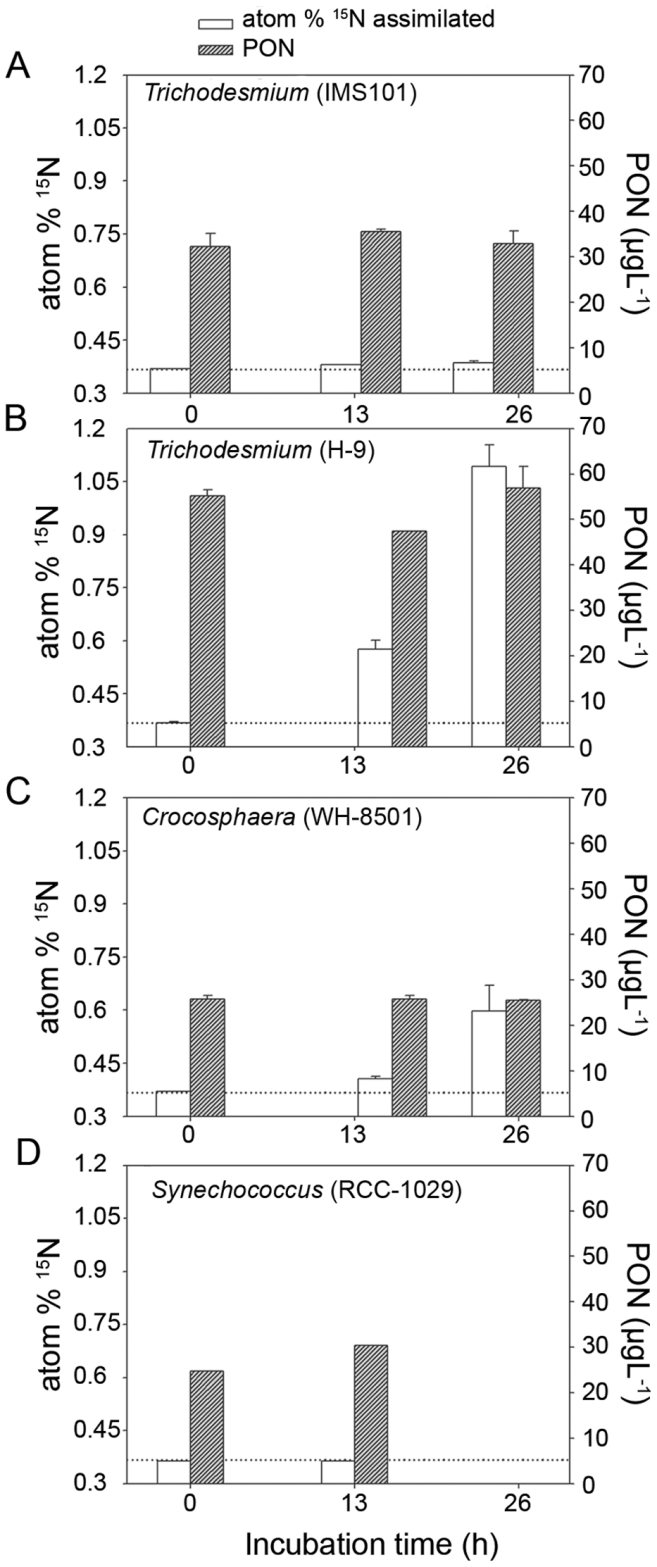
Result of typical time course assimilative N_2_O fixation experiments with a) *Trichodesmium* (IMS101); b) (*Trichodesmium* (H-9); c) *Crocosphaera* (WH-8501); and d) *Synechococcus* (RCC-1029) strains, showing variation in ^15^N enrichment (atom%) and in biomass expressed as PON (µg L^−1^) over incubation time. Dotted horizontal line indicates the value of abundance of ^15^N in PON (∼0.369 atom%).

Several types of experiments were carried out with these cyanobacterial strains (Table S2). All incubated strains except strain RCC 1029 showed a significant excess of ^15^N in PON (referred to as atom%) of 2 to 5 fold higher with respect to the natural abundance of ^15^N in PON (∼0.369 atom%) and its variation seemed to depend on the cell density of each cultured strain (quantified as particulate organic nitrogen “PON”). Time course assays of N_2_O fixation with these strains at different cell densities (measured throughout PON) are illustrated in Figure 3. When observed, the incorporation of ^15^N_2_O increased with incubation time, showing enrichments in the heavier isotope in PON (Fig. 3A, B, C).

**Figure 4:**
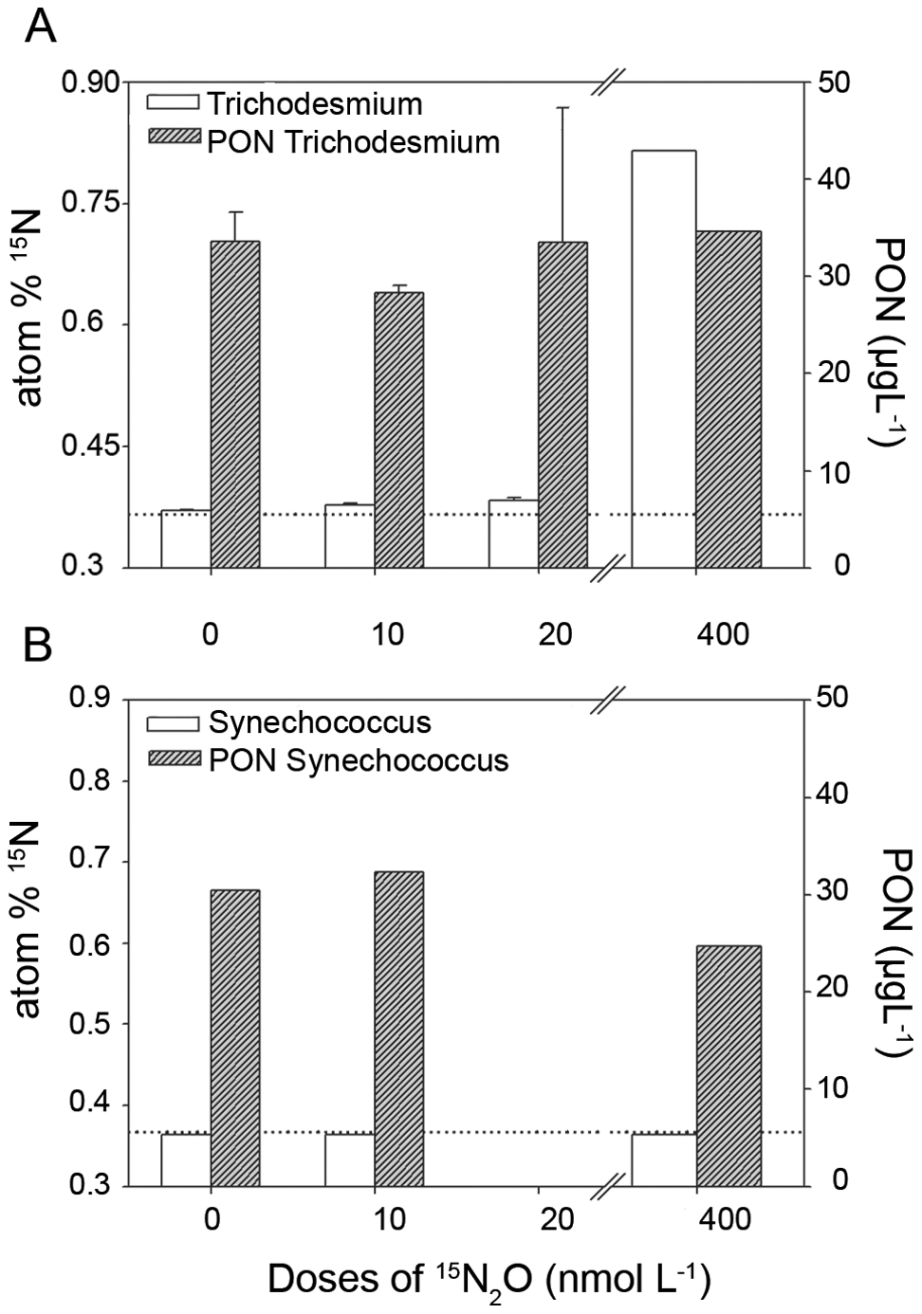
Assimilative N_2_O fixation experiments showing the response of *Trichodesmium* (*IMS101)* and *Synechococcus* (RC 1029) to different doses of dissolved ^15^N_2_O, in increasing order from 10 to 400 nmol L^−1^ of final concentrations. Note change of y-axis scale for experiments.

In addition, different concentrations or doses of dissolved ^15^N_2_O were added to the cultures of *Trichodesmium* (IMS101, Fig. 4A) and *Synechococcus sp*. (RCC 1029, Fig. 4B) in order to verify the nitrogenase kinetics or the dose/response relationship. Whilst *Synechococcus sp.* did not show any ^15^N_2_O incorporation as the doses increased (confirming its inability to use N_2_O), *Trichodesmium* displayed enhanced N_2_O incorporation rates as concentrations of added N_2_O increased from 10–400 nmol L^−1^ final dissolved concentration. This observed trend suggests that nitrogenase has an increasing affinity for N_2_O as N_2_O doses increased (Fig. 4A). It is important to remark that a slight enrichment in atom% (and even atom% excess with respect to natural isotopic abundance) was observed at minimal N_2_O doses (Fig. 4), whose final concentration was close to those environmental levels found in the STG (∼6–10 nmol L^−1^). This suggests that even at very low N_2_O concentrations, N_2_O incorporation may occur. In surface waters associated with coastal upwelling areas, N_2_O concentrations can reach 100 nmol L^−1^ or more [46], and if we look at water under the influence of subsurface O_2_ deficiency, N_2_O concentrations as high as 400 nmol L^−1^ have been reported [46]. Assuming that an increase in atmospheric N_2_O is a likely trend under certain future intensification of eutrophication scenarios [47], and hypoxia and warming [48], this final added concentration of N_2_O in field experiments could be plausible for some marine environments.

Given that some of the N_2_O enrichments used in our field experiments (e.g., the STG Fig. 2A, D, G, J) largely exceeded natural N_2_O levels in the surface ocean by 100 and 1000 times, rates of N_2_O fixation obtained for these sampled areas should be considered as a potential. A simple calculation of the N_2_O turnover time was carried out in surface waters of the STG and CU areas, taking into consideration surface N_2_O inventories and assimilative N_2_O fixation rates 100 and 1000 times smaller than those respectively measured, i.e. around 1 to 10 pmol L^−1^ d^−1^ (Table 2). Estimates showed that the surface N_2_O inventory could be removed entirely between 2 and 12 years. Further kinetic studies and half-saturation constant determinations will be necessary in order to assess the ability of diazotrophic organisms to use N_2_O at its naturally occurring levels in the ocean.

One question that is worth addressing is why N-fixers would fix N_2_O instead of N_2_. When answering this question one has to bear in mind two points: 1) that both pathways are associated with non-selective NifH; and 2) that there might be benefits related to energy for using N_2_O instead of N_2_. The Gibbs free energy required during N_2_O assimilation is thermodynamically advantageous compared to that of N_2_ because the dissociation energy for breaking the N-N bond in the case of N_2_O is only half that required for the N_2_ molecule [49], [50]. Thus, if available, N_2_O may appear to be a more energetically favorable substrate than N_2_.

It is however possible that this process does not directly occur. Therefore, dissimilative reduction of N_2_O to N_2_ followed by the biological fixation of N_2_ into PON may take place, in which case assimilative N_2_O fixation does not constitute a process in itself. This possibility was tested by looking at the isotopic composition of dissolved N_2_ in seawater medium of the cultures (taken thought exetainer) following the addition of ^15^N_2_O to the experiments. The lack of detection of any dissolved ^15^N_2_ in the exetainers after the addition of ^15^N_2_O (during different time incubations) precludes the possibility that N_2_O fixation takes place via N_2_ production and further fixation of N_2_ into PON.

This study provides further arguments in favor of the fact that assimilative N_2_O fixation should exist in marine waters to reduce uncertainties for the marine N cycle. One such argument is that the current isotopic and isotopomeric composition of accumulated atmospheric N_2_O cannot yet be precisely constrained by the N and O isotopic (and isotopomeric) compositions of N_2_O on the ocean surface [51]. A second argument is that it is well-known that there is an imbalance between the sources and sinks of the global N budget [6], [19]. This should become less pronounced if a new N_2_O utilization pathway is included, in some way affecting the global N_2_O cycle [52].

**Figure 5:**
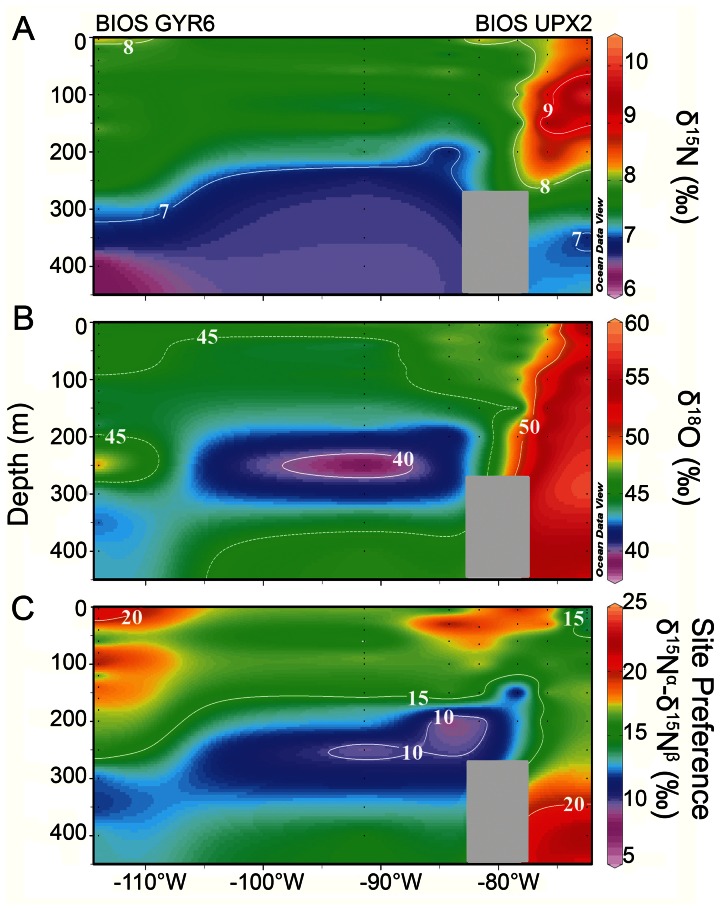
Zonal distribution at 32°S of vertical distributions of N_2_O two-isotope signatures: a) *δ* ^15^N bulk; b) *δ* ^18^O; and c) the site preference (SP = *δ* ^15^N^α^ – *δ* ^15^N^β^). Color scales indicate isotopic composition in ‰. Data interpolation was done with Ocean Data View.

Firstly, the N_2_O dual isotope signatures (*δ* ^15^N^bulk^ and *δ* ^18^O) and their isotopomeric compositions (N^α^N^β^O including the site preference SP = *δ* ^15^N^α^– *δ* ^15^N^β^) have been proposed as a more certain indicator of N_2_O production pathways [51], [53]. The zonal distribution of theses signatures within the ESP is shown in Fig. 5 (Text S2); it reveals important differences in these signatures between the CU and the STG. In the STG, surface values averaged 7.72±0.38, 45.23±1.97 and 16.67‰ (n = 45) for *δ* ^15^N^bulk^, *δ* ^18^O, and SP, respectively (with *δ* ^15^N and *δ* ^18^O referenced to air-N2, and Vienna Standard Mean Ocean Water VSMOW, respectively). On the other hand, the isotopic values in surface waters of the STG were slightly higher than atmospheric values (*δ* ^15^N^bulk^ = 7.0±0.6‰, *δ* ^18^O = 43.7±0.9‰ and SP = 18.70 [51]), and even greater than subsurface water measurements with *δ* ^15^N^bulk^ = 4±1%, *δ* ^18^O = 38.5±3% and SP = 4±4% (see Fig. 5). These surface values at the STG coincide with those reported for the subtropical North and South Pacific gyre [54]–[56], and support the idea that the isotopic N_2_O composition of surface waters cannot simply be the result of mixing between the atmospheric and subsurface marine N_2_O pools.

Therefore, some surface water biological process should operate to maintain the isotopic compositions of *δ* ^15^N, *δ* ^18^O and SP as observed. In view of the prevailing levels of oxygenation in these waters, nitrification would be expected to be the process of greatest importance. In cultures of ammonia-oxidizing bacteria, regardless of the specific mechanism of N_2_O production, this gas is produced as a byproduct with much more depleted isotopic compositions for N and O than those observed in the ocean surface. For example, nitrifying bacterial cultures exhibit average signals of *δ* ^15^N^bulk^ from −54.9‰ to −6.6‰, *δ* ^18^O = 40‰ and a SP ∼33.5‰ via NH_2_OH decomposition, but an SP of about −0.8‰ via nitrifier denitrification [57], [58]. With the recent finding that marine Archaea can produce N_2_O via ammonium oxidation, [4], [59], resulting in a heavier isotopic composition than that observed in ammonia oxidizing bacteria (*δ* ^15^N^bulk^ = 8.7±1.5‰, *δ* ^18^O = 34.0. ±0.9‰ and SP = 30.3±1.2), the current constraints on the global isotopic N_2_O budget are narrowed. However, even when taking into account that N_2_O production in surface water comes from a mix of Bacteria and Archaea as well as the effect of N_2_O influx from the atmosphere (Table S3), observed isotopic compositions of N_2_O in the surface waters of the STG do not match the signal made by N_2_O produced by nitrifiers in the surface water nor the signals observed in either the atmosphere or the subsurface water.

Here, some type of biological process such as assimilative N_2_O reduction into PON that enriches the dissolved N_2_O pool in *δ* ^15^N^bulk^ and *δ* ^18^O is able to resolve the observed discrepancies. This process could also explain the estimated negative values of ΔN_2_O (Fig. 1C) in the STG. However, the negative values of N* and positive values of ΔN_2_O as observed within the CU do not exclude the possibility that assimilative N_2_O fixation is taking place but simply indicate that other N_2_O producing processes (nitrification or partial denitrification), and/or *in situ* total denitrification along with the advection of denitrified waters are occurring faster than assimilative N_2_O fixation, camouflaging both expected N* and ΔN_2_O signals.

Secondly, if N_2_O fixation is significantly taking place in the world’s oceans, it should have an important effect on the global ocean ΔN_2_O inventory which is illustrated in Table S3 and Text S3. As part of assimilative N_2_O fixation appears to be carried by phototrophic diazotrophs, (even if one cannot exclude other micro-organisms), it should be directly linked to diazotroph biomass or abundance. Thus, significant contributions of N_2_O fixation should be observed in these regions where diazotrophs are of quantitative importance. Indeed, emerging patterns of marine N fixation suggest that the Pacific Ocean, in particular the South Pacific (although poorly studied), has the lowest abundance and diazotroph activity compared with other regions such as the North Atlantic, the Indo-Pacific oceanic region and even the Baltic Sea [22].

Further research on N_2_O fixation in other oceanic basins is recommended and several insights appear to indicate that assimilative N_2_O fixation could be higher in regions where diazotrophic microorganisms were more abundant or more active The gathered evidence draws attention to the importance of this pathway for regional and even global N_2_O removal, preventing part of its potential efflux toward the atmosphere. Thus, N_2_O represents a form of yet unreported fixed N that could change our vision and understanding of the oceanic N cycle.

## Methods

This study covers coastal upwelling centers off Peru (CUP ∼8.2–16°S), northern Chile (CUNC, Arica∼ 19°S and Iquique ∼21°S) and central Chile (CUCC, Concepción ∼36.5°S), as well as the eastern subtropical gyre (STG with two transects from the Chilean coast to Eastern Island, covering 20°–27°S and 73°–110°W). The CUP and CUCN areas were visited four times: in October- November 2005 by KN182-9 cruise (R/V Knorr), in February 2007 by the Galathea-3 cruise (R/V Vædderen), in March 2008 by the IQOX cruise (R/V Purihalar), and in November-December 2010 by the Big Rapa cruise (R/V Melville). Biological samples were obtained during oceanographic expeditions in which work in Chilean territorial waters was authorized by the Chilean Government under control of the SHOA (Servicio Hidrográfico y Oceanográfico de la Armada de Chile; www.shoa.cl). Moreover, a Chilean government observer participated in each of the cruises. The area off central Chile was sampled monthly from September 2008 to September 2009 at the COPAS time series station known as St. 18. In addition, unpublished isotopic and isotopomeric N_2_O data from the Biosope cruise (October-December 2004; R/V L’Atalante) was also included (data from Leg 2 from Easter Island to Talcahuano, Chile ∼ 73.1° W, 36.7°S). Table 1 summarizes the location, water depth and other oceanographic variables and parameters measured at the sampling stations.

During all cruises, vertical profiles of temperature, salinity, dissolved O_2_, fluorescence and PAR (Photosynthetically Active Radiation) were obtained using a Conductivity Temperature Depth CTD-O_2_ probe (Sea-Bird Electronics Inc., USA). The O_2_ sensors from the upcast CTD-O were calibrated with discrete samples obtained by Winkler titration (see below). In the case of the KN182-9 cruise, O_2_ sensors were calibrated pre- and post-cruise at Woods Hole Oceanographic Institution (USA). During the Galathea-3 cruise, an ultrasensitive sensor STOX was tested *in situ* [60]. Water column light irradiance, averaged over the visible spectrum (400–700 nm), was measured using a LI-COR (LI-190) quantum sensor, and fluorescence was measured using a WetStar sensor.

Discrete water samples, for chemical analyses and experiments, from the surface (<2 m), down to 400 m were collected using Niskin bottles (12 L) attached to a rosette sampler. Core parameters, including dissolved O_2_ and N_2_O, nutrients (NH_4_^+^, NO_3_^−^, NO_2_^−^, PO_4_^3−^), Chl-a, particulate organic carbon and nitrogen (POC and PON), and their natural C and N isotopic compositions, were determined at all stations. N*, a quasi-conservative tracer defined as a linear combination of NO_3_^−^ and PO_4_^3−^, was estimated from nutrient concentrations throughout the water column [21]. Apparent N_2_O production (ΔN_2_O) was obtained from the difference between the N_2_O saturation at equilibrium with the atmosphere and its concentration measured in seawater [61].

In terms of the employed analytical methods, dissolved O_2_ was analyzed in triplicate by automatic Winkler titration. The samples for N_2_O analyses were transferred directly into 20-mL glass vials (triplicates), preserved with 50 µL of saturated HgCl_2_ and sealed with butyl rubber and aluminum cap stoppers. N_2_O was determined by Helium equilibration in the vial, followed by quantification with a Varian 3380 Gas Chromatograph (GC) using an electron capture detector maintained at 350°C, for more details see [46]. A calibration curve was made with 5 points (He, 0.1 ppm, air, 0.5 ppm, and 1 ppm) and the detector linearly responded to this concentration range. The analytical error for the N_2_O analysis was less than 3%.

Nutrient samples were collected with a 60 mL plastic syringe and filtered through a glass fiber filter (pore size 0.7 µm) into high-density polypropylene scintillation vials. Samples were stored at –20°C until laboratory analysis, except during the KN182-9 and Galathea-3 cruises when dissolved NO_2_^−^ and PO_4_^−3^ concentrations were immediately determined on board [62]. For determination of Chl-a, a fluorometry method was used for filtered seawater through a 45 mm Whatman GF/F filter [63]. Analyses of particulate organic C and N (POC and PON) and their natural ^13^C and ^15^N isotopic compositions were carried out after filtering 1 L of seawater through pre-combusted 0.7 µm glass fiber filters (22 mm Whatman *GF-F*) and stored at –20°C until analysis. Filters were dried at 60°C for 12 h before determining their isotopic composition via continuous-flow isotope ratio mass spectrometry (IRMS; Finnigan Delta Plus). Reproducibility for ^13^C and ^15^N was greater than 0.11‰ and 0.02‰, respectively, based on the acetanilide standard used as reference material. Isotope ratios were expressed as per mil deviations from the isotopic composition of Vienna PDB and air, for ^13^C and ^15^N, respectively [64]. Significant differences were checked between the enrichment as ^15^N atom% and its atom% excess of PON in the experiments with respect to the natural background or natural isotopic composition of PON taken at each sampled station and depth.

Additionally, oceanographic/meteorological and biogeochemical variables/parameters shown in Table 1 were measured and estimated. The mixed layer depth (Z_m_) was obtained from vertical density profiles measured every 1 dbar using the CTD sensor, and the depth of the euphotic zone (irradiation at 1% of its surface value) was estimated from the attenuation coefficient of downwelling irradiance averaged over the visible spectrum (400–700 nm) measured by a LI-COR sensor. In the few instances where light profiling was not possible (night sampling), light profiles were estimated using surface irradiation (assumed 4% surface reflection) and the vertical attenuation coefficient of PAR (K) from the previous day.

In order to detect differences in N_2_O fixation rates among the sampled areas (i.e., STG, CUP, CUNC and CUCC), non parametric Kruskal Wallis test was carried out using statistical language R [65]. Additionally, a multiple linear regression model was performed to assess the variables that determine the variance of N_2_O fixation rates, with a prior logarithmic transformation of this dependent variable [65]. Categorical variables associated with the different study areas were also included. A step-wise selection was used to test the significance of each variable in the model. The best model was obtained by determining its heteroscedasticity and p-value (p<0.05). Models were then contrasted using the Akaike Information Criteria (AIC). The comparison among light (65, 30, 4 and 1% of irradiance) and dark treatments for N_2_O fixation rates was done with a Mann–Whitney test.

### N_2_O Fixation Experiments (Assimilative N_2_O Reduction into PON)

Experiments for assimilative N_2_O fixation were performed using an improved stable isotope technique [66] at selected stations listed in Table 1. ^15^N-labeled N_2_O gas (99 Atom %; CAMPRO SCIENTIFIC) was offered as a substrate during the experiments to measure N_2_O fixation rates by incubating samples. Assimilative N_2_O fixation rates were assayed with both field samples and cultured cyanobacteria strains, both subjected to different experimental treatments (see Table S2).

Field samples were incubated on board, under temperature-controlled conditions, using an *in situ* range of light intensities, as well as dark conditions. For this purpose, seawater was dispensed from Niskin bottles using a gas-tight Tygon tube, to avoid any oxygenation, into 1.5–2 L double-laminated aluminum-polyethylene or transparent Tedlar® bags. The volume and weight of the filled bags were controlled at the beginning and end of the incubation process, and real volumes were used in rate calculations. As an additional precaution, a permeability test was performed on bags prior to the experiments. The bags were filled with pure helium and monitored for 24 h using gas chromatography. They showed no atmospheric gas (N_2_O or CH_4_) intrusions, thus ensuring hermetically sealed conditions. Atmospheric O_2_ could not be tested in the bags via chromatography (given the sensitivity of the chromatographic method), however tests were performed using atmospheric N_2_O as a tracer in vacuum emptied bags. As N_2_O intrusions were not detected inside the bag during the days following the sampling, O_2_ intrusions during incubations in similar bags were considered unlikely. Each bag had a hose/valve with a septum through which ^15^N_2_O tracer and different treatments, i.e., ^15^N_2_O, HgCl_2_ (see Table S2) were injected using gastight syringes. Tracer addition was carried out at a final concentration of 10∼20 µmol L^−1^ (1 or 2 mL of tracer gas into 1.5–2 L incubation volume). The relative tracer (^15^N_2_O) concentration with respect to natural ^14^N_2_O background varied between 100 and 1000 fold (depending on seawater N_2_O levels), taking into consideration solubility and partition coefficients, as well as the ratio between gas and liquid phases of N_2_O in the bag [61].

Most of the incubations were performed using six deck incubators maintained at sea surface temperature and with light intensities ranging between 65% and 4% of incident light (Lee Filters®). Samples from below the base of the euphotic layer were incubated in the dark in a thermo-regulated bath (Johnson Control®, KN182-9 cruise) or a temperature controlled incubator at temperatures close to *in situ*. During the Big Rapa cruise, surface duplicate samples were simultaneously incubated under *in situ* light and dark conditions. The incubations lasted 24 h and they were terminated by gentle filtration onto pre-combusted 0.7 µm glass fiber filters (Whatman GF/F filters) using a vacuum (<100 mm Hg) or a peristaltic pump. Filters were stored at −20°C until laboratory analysis. Off central Chile, samples were incubated at two times (t = 12 and t = 24) with artificial light in a temperature-controlled room.

With samples obtained off central Chile (see above) and with cyanobacterial strains cultivated in the laboratory, assimilative N_2_O fixation rates (several batches) were assayed as time course experiments. In order to achieve this, samples (triplicates) were amended with ^15^N_2_O and incubated for 12 and 24h. Incubations were terminated by filtration as was outlined above. To assay assimilative N_2_O fixation,^ 15^N_2_O was offered as a substrate during the experiments with *Trichodesmium, Croccosphaera* and *Synechococcus*. These diazotrophic cyanobacteria were isolated by Jon Waterbury at WHOI and correspond to *Trichodesmium erythraeum*, strain IMS101 (http://img.jgi.doe.gov/cgi-bin/w/); *Trichodesmium sp*., strain H9-4 (genetically H9 looks like *T. tenue*); and *Crocosphaera watsonii*, strain WH-8501 (http://genome.jgi-psf.org/crowa/crowa). Additionally, the *Synechococcus sp*. Biosope_141 D strain RCC 1029 was obtained from the Rosscof collection (http://www.sb-roscoff.fr/Phyto/RCC/index.php).

The diazotrophic strains were cultivated at 24°C under artificial light according to a daily cycle of 12 h light/12 h dark, in YBCII artificial seawater medium with no nitrogen sources [67], while *Synechococcus* was cultivated with the same cycle in PCR-S11 medium [68] at 18°C. Several batches of these strains were then transferred to GC bottles (50 or 125 mL), and 50 or 100 µL of ^15^N_2_O (depending on of the vials) was injected into each bottle through septa. Different doses were inoculated, with final concentrations going from 10 to 400 nmol L^−1^ (expected levels in the study area). Cell density was variable depending on the type of cultivated strain and date when experiments were undertaken. PON level (µg L^−1^) measured during each time incubation was used as a biomass index. No variations of PON (Fig. 3) were recorded during time course experiments, indicating no net growth during incubations.

Prior to each experiment and tracer inoculation, the health and purity of cultures were checked using flow cytometry and microscopy. The IMS101, H-9, WH-8501, RCC-1029 strains were not axenic cultures and, therefore, bacterial cell density was periodically checked in the cultures with a FACSCalibur flow cytometer equipped with an ion–argon laser of 488 nm of 15 mW (Becton Dickinson). The picoplanktonic bacterial abundance was estimated from samples previously stained with SYBR-Green I (10,000 x; Molecular Probes) following Marie *et al*, 2000 [69]. Cell Quest Pro and Cytow software were used for data acquisition and analysis. In the case of WH-8501, abundances varied from 5×10^3^ to ∼ 4×10^4^ cell mL^−1^, but the abundance of WH-8501strain was an order of greater magnitude.

Finally, after addition of ^15^N_2_O to selected experiments with H-9 and WH-8501 strains, liquid samples were collected in exetainers (LABCO) with particular care to avoid air contamination, and the isotopic composition of N_2_ was measured in order to check if one-step reaction transforming N_2_O directly into NH_4_^+^ proceeded without intermediary N_2_. The isotope ratios of N_2_ were measured in gas mixtures (headspace) using a Thermo Finnigan GasBench+PreCon trace gas concentration system interfaced to a Thermo Scientific Delta V Plus isotope-ratio mass spectrometer (U Davis, USA; http://stableisotopefacility.ucdavis.edu/n2.html).

## Supporting Information

Table S1 Oceanic N_2_O undersaturation (saturation %) and air-sea flux (µmol m^−2^ d^−1^) reported in surface and subsurface/intermediate waters around the global ocean. Depths of hypoxic/suboxic waters are indicted
(DOCX)

Table S2 Experimental setup used with in natural and cyanobacteria cultured samples to asses assimilative N_2_O fixation.
(DOCX)

Table S3 ΔN_2_O inventories (µmol·m^−2^) and estimated net N_2_O production (µmol·m^−2^·d^−1^) in surface waters at selected stations along N_2_O air-sea exchange (µmol·m^−2^·d^−1^) and N_2_O consumption rates by fixation. In some station, denitrification and N_2_ fixation rates (both based on published data) are available in order to compare with other N_2_O consuming processes.
(DOCX)

Text S1 References Table S1
(DOCX)

Text S2 Isotopic and Isotopomeric determination
(DOCX)

Text S3 N_2_O mass balance
(DOCX)
