# Supplementary material for: Biological N2O Fixation in the Eastern South Pacific Ocean and Marine Cyanobacterial Cultures
Source: PLoS One. 2013 May 23;8(5):e63956. doi: 10.1371/journal.pone.0063956 (PMC3662754; doi:10.1371/journal.pone.0063956)
Supplement: Table S1 — Oceanic N2O undersaturation (saturation %) and air-sea flux (µmol m−2 d−1) reported in surface and subsurface/intermediate waters around the global ocean. Depths of hypoxic/suboxic waters are indicted (DOCX) [file pone.0063956.s001.docx]

| Table S1. Oceanic N_2_O undersaturation (saturation %) and air-sea flux (μmol m^-2^ d^-^**^1^**) reported in surface and subsurface/intermediate waters around the global ocean. Depths of hypoxic/suboxic waters are indicted | | | | |
| --- | --- | --- | --- | --- |
|  | | | | |
| **Region** | **N_2_O Saturation**  **(%)** | **Fluxes**  **(μmol m^-2^ d^-1^)** | **Depth**  **(m)** | **References** |
| North Atlantic Drift | 98±10 | -0.04 – 0.08 | surface | [70] |
| North Atlantic Tropical gyre | 97±4 – 104±3 | -0.02 – 0.87 | surface | [70] |
| Puerto Rico (Parguera) | 59 – 424.85 | nr | surface | [71] |
| Eastern Equatorial Pacific | 74.5 – 125.9 | -0.9±0.7 | surface | [18] |
| Arabian Sea (A9) | 97±4 – 204±16 | 1.6 – 5.1 | surface | [72] |
| Arabian Sea (A4) | 94±9 – 154±1 | 1.6 – 5.1 | surface | [72] |
| Baltic Sea | 92.5 – 312 | 0.3 – 6.22 | surface | [73] |
| Southwest of Australia | Weakly undersaturated | nr | surface | [74] |
| Central Pacific gyre | Slightly undersaturated | 0.04 – 5.2 | surface | [74] |
| Baltic Sea | 79.3±10.7^†^ | nr | surface | [75] |
| Baltic Sea | 79 | nr | surface | [76] |
| Antartic circumpolar current | 98 – 104 | -1.18 – 1.75 | surface | [77] |
| East China Sea | 94 – 382 | -0.69 – 97.48 | surface | [78] |
| Northwest Black Sea | 96 – 149 | 1.6 – 5.2 | surface | [79] |
| Eastern Tropical North Pacific | 95 | nr | surface | [80] |
| North Atlantic | 97 – 118 | 0.6±0.95 | surface | [81] |
| Subtropical gyre ESP | 91 – 99 | -0.48 ±0.44 | surface | [16] |
| Coastal upwelling region | 78-99^ψ^ | -0.33±1.4 | surface | [46] |
| Tropical North Pacific | >50 | nr | 150 – 800 | [80] |
| Peruvian Upwelling | <40 | nr | 150 – 250 | [6] |
| Cariaco Trench | <20 | nr | <300 | [82] |
| Black Sea | ~0 | nr | >70 | [83] |
| Arabian Sea | ~60 | nr | 200 – 500 | [84] |
| Arabian and Indian Sea | <80 | nr | 250 – 500 | [85, 86, 87] |
| Northern Chile  Peruvian Upwelling | <60 | nr | 100 – 250 | [39, 88] |
| Surface denotes waters come from the mixed layer depth (MLD). nr.: not reported ; ψ: measured during austral summer; †: data averaged | | | | |
