# Supplementary material for: Biological N2O Fixation in the Eastern South Pacific Ocean and Marine Cyanobacterial Cultures
Source: PLoS One. 2013 May 23;8(5):e63956. doi: 10.1371/journal.pone.0063956 (PMC3662754; doi:10.1371/journal.pone.0063956)
Supplement: Table S2 — Experimental setup used with in natural and cyanobacteria cultured samples to asses assimilative N2O fixation. (DOCX) [file pone.0063956.s002.docx]

| Table S2. Experimental setup used with in natural and cyanobacteria cultured samples to asses assimilative N_2_O fixation.. | | | | |
| --- | --- | --- | --- | --- |
|  | | | | |
| **Type the sample** | **Isotope and dose** | **Treatment** | **Purpose of the experiment** | **Comments** |
| Environmental samples | ^15^N_2_O ~ 10/20  µmol L^-1^ | Simulation of *in n - situ* light or dark condition | Biological fixation  (^15^N- enrichment on PON | Samples came from different areas of the ESP |
| Environmental  samples | ^15^N_2_O 10 µmol L^-1^ | Simultaneous incubation (duplicate samples) under light and dark condition | Dependency of light condition | Samples come from Big Rapa cruise. |
| Environmental  samples | ^15^N_2_O 20 µmol L^-1^ | Saturated HgCl_2_ | Non biological fixation | 1 mL of Saturated HgCl_2_ was added to the bag |
| *H-9* and *W 8501*strains | ^15^N_2_O 10 µmol L^-1^ | Light condition | Recover ^15^N_2_ gas  Direct or non-direct pathway | Lab. conditions |
| *H-9, ISM 101,*  *W 8501* and *RCC 1029* strains | ^15^N_2_O 10 µmol L^-1^ | Time course experiments  (two times) | Linear ^15^N enrichment response | Lab. conditions |
| *ISM 101* strain | ^15^N_2_O 10 µmol L^-1^ | Simultaneous light and dark condition | Dependency of Light | Artificial light condition |
| *RCC 1029* strain | ^15^N_2_O from 10 to 400 nmol L^-1^ | Increasing doses | Kinetic response | Artificial light condition |
| *ISM 101* strain | ^15^N_2_O from 10 to 400 nmol L^-1^ | Increasing doses | Kinetic response | Artificial light conditions |
|  | | | | |
