# Supplementary material for: Biological N2O Fixation in the Eastern South Pacific Ocean and Marine Cyanobacterial Cultures
Source: PLoS One. 2013 May 23;8(5):e63956. doi: 10.1371/journal.pone.0063956 (PMC3662754; doi:10.1371/journal.pone.0063956)
Supplement: Table S3 — ΔN2O inventories (µmol·m−2) and estimated net N2O production (µmol·m−2·d−1) in surface waters at selected stations along N2O air-sea exchange (µmol·m−2·d−1) and N2O consumption rates by fixation. In some station, denitrification and N2 fixation rates (both based on published data) are available in order to compare with other N2O consuming processes. (DOCX) [file pone.0063956.s003.docx]

| Table S3. ΔN_2_O inventories (µmol·m^-2^) and estimated net N_2_O production (µmol·m^-2^·d^-1^) in surface waters at selected stations along N_2_O air-sea exchange (µmol·m^-2^·d^-1^) and N_2_O consumption rates by fixation. In some station, denitrification and N_2_ fixation rates (both based on published data) are available in order to compare with other N_2_O consuming processes. | | | | | | |
| --- | --- | --- | --- | --- | --- | --- |
|  | | | | | | |
| **Areas** | **Station†** | **Whole‡ Inventory ΔN_2_O (µmol·m^-2^)** | **Surface† Inventory ΔN_2_O (µmol·m^-2^)** | **Surface^§^ N_2_O production (µmol·m^-2^·d^-1^)** | **Surface† N_2_O fixation rate (µmol·m^-2^·d^-1^)** | **Air-sea N_2_O flux^Ѓ^ (µmol·m^-2^·d^-1^)** |
| Coastal Upwelling | G04 * | 1,244 | 325.9. | 259.9 | -160±245 | -12.8 |
|  | G05 * | 2,515 | 190.1 | 41.72 | -0.84±0.11 | -0.61 |
|  | G09 * | 2,438 | 732.7 | 434.6 | -4.03±3.47 | -55.3 |
|  | G11 * | 6,187 | 1416 | 880.0 | -1.69±2.06 | -60.0 |
|  | G16* | 5,582 | 1733 | 230.0 | -2.07±0.18 | -100.6 |
|  | G17 * | 4,777 | 893.2 | 411.3 | -0.385±0.356 | -29.4 |
|  | G22 * | 4,850 | 779.2 | 128.8 | -1.56±0.43 | -26.1 |
|  | BR-1** | 5,399 | 47.64 | 37.53 | -6.77±11.2 | -10.09 |
| Subtropical Gyre | BR-7** | 1,160 | -37.27 | -6.60 | -10.40±5.56 | +0.21 |
|  | BIOS-GYR** | 408.4 | -57,81 | -5.17 | nm | +0.35 |
|  | BIOS-EGY** | 1,264 | - 93,68 | -37.81 | nm | -0.52 |
| Negative or positive sign indicates N_2_O removal or addition process, respectively. Note that for the STG stations N_2_O influx were measured (i.e., from the atmosphere to the surface waters)†Station selected according to complete data available; ‡ inventory estimated from data integrated between the surface and 400 m depth; †Inventories estimated from data integrated between the surface and 30 m depth (in the case of CU stations) and 120 m (in the case of STG stations) taking into consideration averaged Z_m_; ^§^Surface net N_2_O production/consumption based on inventory and piston velocity using the same integration criteria. * Denotes the station where denitrification and N_2_ fixation were simultaneously detected in independent way (based on published data). ** Denotes that N_2_ fixation rates are available. nm: not measured. | | | | | | |
