# Supplementary material for: Biological N2O Fixation in the Eastern South Pacific Ocean and Marine Cyanobacterial Cultures
Source: PLoS One. 2013 May 23;8(5):e63956. doi: 10.1371/journal.pone.0063956 (PMC3662754; doi:10.1371/journal.pone.0063956)
Supplement: Text S1 — References Table S1 (DOCX) [file pone.0063956.s004.docx]

Text S1

References Table S1

70. Forster G, Upstill-Goddard RC, Gist N, Robinson C, Uher G, et al. (2009) Nitrous oxide and methane in the Atlantic Ocean between 50°N and 52°S: Latitudinal distribution and sea-to-air flux. Deep-Sea Res Pt II 56: 964-976. doi: 10.1016/j.dsr2.2008.12.002. Available: <http://www.sciencedirect.com/science/article/pii/S0967064508004116>. Accessed 2013 April 23.

71. Aguilera A, Corredor JE, Morell JM (1992) Oxido Nitroso en el Mar Caribe Nororiental. Caribbean Journal of Science 28: 70-80. doi:oclc/23165651. <http://www.researchgate.net/publication/35808649_Oxido_nitroso_en_el_Mar_Caribe_Nororiental_>. Accessed 2013 April 23.

72. Upstill-Goddard RC, Barnes J, Owens NJP (1999) Nitrous oxide and methane during the 1994 SW monsoon in the Arabian Sea/northwestern Indian Ocean. J Geophys Res 104: 30067-30084. doi: 10.1029/1999jc900232. Available: <http://dx.doi.org/10.1029/1999JC900232> .Accessed 2013 April 23.

73. Bange HW, Dahlke S, Ramesh R, Meyer-Reil LA, Rapsomanikis S, et al. (1998) Seasonal Study of Methane and Nitrous Oxide in the Coastal Waters of the Southern Baltic Sea. Estuar Coast Shelf S 47: 807-817. doi:10.1006/ecss.1998.0397. Available:<http://www.researchgate.net/publication/223277148_Seasonal_Study_of_Methane_and_Nitrous_Oxide_in_the_Coastal_Waters_of_the_Southern_Baltic_Sea>.Accessed 2013 April 23.

74. Butler JH, Elkins JW, Thompson TM, Egan KB (1989) Tropospheric and Dissolved N_2_O of the West Pacific and East Indian Oceans During the El Niño Southern Oscillation Event of 1987. J Geophys Res 94: 14865-14877. doi: 10.1029/JD094iD12p14865. Available: <http://dx.doi.org/10.1029/JD094iD12p14865>.Accessed 2013 April 23.

75. Walter S, Breitenbach U, Bange HW, Nausch G, Wallace DWR (2006) Distribution of N_2_O in the Baltic Sea during transition from anoxic to oxic conditions. Biogeosciences 3: 557-570. Available: <http://www.biogeosciences.net/3/557/2006/> .Accessed 2013 April 23.

76. Rönner U (1983) Distribution, production and consumption of nitrous oxide in the Baltic Sea. Geochim Cosmochim Acta 47: 2179-2188. doi: 10.1016/0016-7037(83)90041-8. Available:<http://www.sciencedirect.com/science/article/pii/0016703783900418>.Accessed 2013 April 23.

77. Law CS, Ling RD (2001) Nitrous oxide flux and response to increased iron availability in the Antarctic Circumpolar Current. Deep-Sea Res Pt II 48: 2509-2527. doi: 10.1016/S0967-0645(01)00006-6. Available:<http://www.sciencedirect.com/science/article/pii/S0967064501000066> .Accessed 2013 April 23.

78. Zhang G, Zhang J, Ren J, Li J, Liu S (2008) Distributions and sea-to-air fluxes of methane and nitrous oxide in the North East China Sea in summer. Mar Chem 110: 42-55. doi: 10.1016/j.marchem.2008.02.005 Available: <http://www.sciencedirect.com/science/article/pii/S0304420308000145>.Accessed 2013 April 23.

79. Amouroux D, Roberts G, Rapsomanikis S, Andreae MO (2002) Biogenic Gas (CH_4_, N_2_O, DMS) Emission to the Atmosphere from Near-shore and Shelf Waters of the North-western Black Sea. Estuar Coast Shelf S 54: 575-587.doi: 10.1006/ecss.2000.0666. Available: <http://www.sciencedirect.com/science/article/pii/S0272771400906668>. Accessed 2013 April 23.

80. Cohen Y, Gordon LI (1978) Nitrous oxide in the oxygen minimum of the eastern tropical North Pacific: evidence for its consumption during denitrification and possible mechanisms for its production. Deep-Sea Res 25: 509-524.doi: 10.1016/0146-6291(78)90640-9. Available: <http://www.sciencedirect.com/science/article/pii/0146629178906409>.Accessed 2013 April 23.

81. Walter S, Bange HW, Wallace DWR (2004) Nitrous oxide in the surface layer of the tropical North Atlantic Ocean along a west to east transec. Geophys Res Lett 31: L23S07. doi: 10.1029/2004gl019937. Available: <http://dx.doi.org/10.1029/2004GL019937>.Accessed 2013 April 23.

82. Hashimoto LK, Kaplan WA, Wofsy SC, McElroy MB (1983) Transformations of fixed nitrogen and N_2_O in the Cariaco Trench. Deep-Sea Res 30: 575-590. doi: 10.1016/0198-0149(83)90037-7. Available:<http://www.sciencedirect.com/science/article/pii/0198014983900377>.Accessed 2013 April 23.

83. Westley MB, Yamagishi H, Popp BN, Yoshida N (2006) Nitrous oxide cycling in the Black Sea inferred from stable isotope and isotopomer distributions. Deep-Sea Res Pt II 53: 1802-1816. doi: 10.1016/j.dsr2.2006.03.012. Available:<http://www.sciencedirect.com/science/article/pii/S0967064506001482>.Accessed 2013 April 23.

84. Bange HW, Rapsomanikis S, Andreae MO (2001) Nitrous oxide cycling in the Arabian Sea. J Geophys Res 106: 1053-1065. doi: 10.1029/1999jc000284.Available: <http://dx.doi.org/10.1029/1999JC000284>.Accessed 2013 April 23.

85. Naqvi SWA (1987) Some aspects of the oxygen-deficient conditions and denitrification in the Arabian Sea. J Mar Res 45: 049-1072. **doi:**<http://dx.doi.org/10.1357/002224087788327118>.Available:<http://www.ingentaconnect.com/content/jmr/jmr/1987/00000045/00000004/art00010?token=004912a3ae573d257025702c236e462a40632d5b20667c4e7547543c7e386f642f466f8d8>.Accessed 2013 April 23.

86. Naqvi SWA, Noronha RJ (1991) Nitrous Oxide in the Arabian Sea. Deep-Sea Res 38: 871-890. doi: [http://dx.doi.org/10.1016/0198-0149(91)90023-9](http://dx.doi.org/10.1016/0198-0149%2891%2990023-9).Available: <http://www.sciencedirect.com/science/article/pii/0198014991900239>. Accessed 2013 April 23.

87. Naqvi SWA, Bange HW, Farías L, Monteiro PMS, Scranton MI, et al. (2010) Marine hypoxia/anoxia as a source of CH_4_ and N_2_O. Biogeosciences 7: 2159-2190. doi: 10.5194/bg-7-2159-2010. Available: <http://www.biogeosciences.net/7/2159/2010/bg-7-2159-2010.html>. Accessed 2013 April 23.

88. Farias L, Paulmier A, Gallegos M (2007) Nitrous oxide and N-nutrient cycling in the oxygen minimum zone off northern Chile. Deep-Sea Res Pt I 54: 164-180. doi: 10.1016/j.dsr.2006.11.003. Available:<http://www.sciencedirect.com/science/article/pii/S0967063706003098>. Accessed 2013 April 23.
