# Supplementary material for: Biological N2O Fixation in the Eastern South Pacific Ocean and Marine Cyanobacterial Cultures
Source: PLoS One. 2013 May 23;8(5):e63956. doi: 10.1371/journal.pone.0063956 (PMC3662754; doi:10.1371/journal.pone.0063956)
Supplement: Text S2 — Isotopic and Isotopomeric determination (DOCX) [file pone.0063956.s005.docx]

Text S2

Isotopic and Isotopomeric determination

Data for of isotopic and isotopomeric compositionsN_2_O were obtained during Biosope cruise. In this cruise, seawater was transferred directly into 125-mL glass flasks (duplicate), preserved with HgCl_2_, and sealed with butyl rubber stoppers. Isotopic and isotopomeric determinations were carried out at the Tokyo Institute of Technology using a Finnigan MAT 252 mass spectrometer [94]. For this, N_2_O was extracted from samples by sparging with Helium and introduced into a pre-concentration-gas chromatography-isotopic ratio mass spectrometry system. The signals of δ^15^N_bulk_ and δ^18^O in N_2_O were determined, as a function of atmospheric nitrogen and VSMOW, respectively. N_2_O isotopomers were determined based on the analysis of ionic mass fragments (NO^+^ and N_2_O^+^) formed by the electron impact of N_2_O. This determination is possible given that NO^+^ fragments contain the central nitrogen (α), which allows the conversion of the fragment ratios into isotopic ratios of ^14^N^15^NO and ^15^N^14^NO. Although there is a rearrangement reaction during the ionic fragmentation process, its magnitude can be determined and used for correction. The precision of these measurements is typically better than 0.5‰ for δ^15^N_bulk_ and δ^18^O, and better than 1‰ for δ^15^N^α^ and δ^15^N^β^. Furthermore, the definition of δ^15^N_bulk_ = (δ^15^N^α^+ δ^15^N^β^)/2 allowed us to compare the relative abundance of isotopomers α and β with the relative isotopic abundance of ^15^N [51].

${}^{15}R^{\alpha}=\frac{\left[ {}^{14}N{}^{15}N{}^{16}O \right]}{\left[ {}^{14}N{}^{14}N{}^{16}O \right]}$ (4)

${}^{15}R^{\beta}=\frac{\left[ {}^{15}N{}^{14}N{}^{16}O \right]}{\left[ {}^{14}N{}^{14}N{}^{16}O \right]}$ (5)

$\delta^{15}R^{\alpha}=\frac{\left[ {}^{15}R^{\alpha} \right]}{\left[ {}^{15}R^{\alpha}\left( std \right)-1 \right]}*1000$ (6)

$\delta^{15}R^{\beta}=\frac{\left[ {}^{15}R^{\beta} \right]}{\left[ {}^{15}R^{\beta}\left( std \right)-1 \right]}*1000$ (7)

Regarding the isotopic values particularly observed at the surface waters of the STG, a biological process that enriches the residual N_2_O pool in *δ*^15^N^bulk^ and *δ*^18^O is required. The use of N_2_O during assimilative reduction should produce a similar effect to canonical denitrification [95] and could be consistent with the high values of *δ*^18^O and *δ*^15^N^bulk^ of N_2_O observed in subtropical surface waters relative to subsurface waters or the atmosphere.

References

94. Toyoda S, Yoshida N (1999) Determination of Nitrogen Isotopomers of Nitrous Oxide on a Modified Isotope Ratio Mass Spectrometer. Anal Chem 71: 4711-4718.doi: 10.1021/ac9904563. Available: <http://dx.doi.org/10.1021/ac9904563>.Accessed 2013 April 23.

95. Yoshinari T, Altabet MA, Naqvi SWA, Codispoti L, Jayakumar A, et al. (1997) Nitrogen and oxygen isotopic composition of N_2_O from suboxic waters of the eastern tropical North Pacific and the Arabian Sea measurement by continuous-flow isotope-ratio monitoring. Mar Chem 56: 253-264. doi: 10.1016/S0304-4203(96)00073-4. Available: <http://www.sciencedirect.com/science/article/pii/S0304420396000734>.Accessed 2013 April 23.
