# Supplementary material for: Biological N2O Fixation in the Eastern South Pacific Ocean and Marine Cyanobacterial Cultures
Source: PLoS One. 2013 May 23;8(5):e63956. doi: 10.1371/journal.pone.0063956 (PMC3662754; doi:10.1371/journal.pone.0063956)
Supplement: Text S3 — N2O mass balance (DOCX) [file pone.0063956.s006.docx]

Text S3:

N_2_O mass balance

In order to interpret the impact of assimilative N_2_O fixation on the ΔN_2_O inventory, a N_2_O mass balance was made as an exercise to elucidate processes responsible for adding or removing N_2_O from the mixed layer (Z_m,_ Table 1). The thickness of the Z_m_ always includes the photic zone and for comparative analyses (inventories), the Z_m_ averaged 30 m at the CU, and 120 m depth at STG. Integrated ΔN_2_O inventories and N_2_O fixation rates in the surface water of the study area were calculated by numerically integrating (trapezoidal quadrate) over specific depth ranges (0-30 m for CU or 0-120 m depth for STG). For N_2_O fixation estimates, samples incubated from 65 to the 4% surface irradiance depth were included.

Assuming a steady state of N_2_O concentrations over time in surface water (no accumulation of N_2_O) and that piston velocity is the main physical mechanism by which a gas is pushed throughout the surface layer, N_2_O production rates in the surface layer were calculated as the integrated ΔN_2_O inventory multiplied by the estimated piston velocity (see below) from each station at the moment of sampling. Daily rates were expressed as µmol m^-2^. Very similar N_2_O production rates (up to 20% of variation) were obtained, in the subtropical Pacific gyre [16], through a mass balance with outward (through the air-sea interface) and inward (through the pycnocline) N_2_O fluxes from and to the mixing layer, taking into account turbulent diffusion and vertical advection [16]. This confirms the validity of comparing N_2_O production with other N_2_O inputs/outputs (N_2_O flux).

Air-sea N_2_O fluxes *F_air_* (μmol day^-1^ m^-2^) were estimated, using the following relationship:

$F_{air}=k_{w}\left( C_{w}-C_{sat} \right)$, (1)

where *k_w_* (m s^-1^) is the gas transfer velocity dependent on wind speed; *C_w_* is the N_2_O concentration (nmol L^-1^); and *C_sat_* is the N_2_O concentration at relative equilibrium with the atmospheric concentration according to the solubility parameterization [89]. The calculation of *k*_w_ was done using *Nightingale’s* parameterization[89].

$k_{w}=\left( 9.25*{10}^{-7}*u+6.17*{10}^{-7}*u^{2} \right)\left( \frac{Sc}{600} \right)^{-0.5}$, (2)

where *u* is the wind speed (m s^-1^) and *Sc* is the Schmidt number for N_2_O, i.e., the relationship between viscosity and the diffusion coefficient of N_2_O in water, dependent on seawater temperature and salinity. For N_2_O, the Schmidt number as a function of temperature (T in ºC) was estimated [90].

$Sc=2301.1-151.15*T+4.7364*T^{2}-0.059431*T^{3}$, (3)

The wind speed was measured on board (KN182-9, Galathea-3 and Big Rapa cruises) and normalized to 10 m height [91]. Thus piston velocities in this study ranged from 1.4 *10^-5^ to 7.08 * 10^-5^ m sec^-1^.

Table S3 shows, for selected stations, estimated N_2_O production in the surface layer along with air-sea flux that adds or removes N_2_O depending on the direction and integrated N_2_O fixation rates. The later rates explain between 0.2–60% of the potential N_2_O removal processes, consuming their inventories in periods ranging from about 2 to 2000 days (Table S3). A N_2_O removal process (as biological N_2_O fixation) must be particularly necessary in oceanic gyres, where there is an inward flux of N_2_O from the atmosphere, as was also observed by[16] and has been reported in other oceanic areas (Table S1). In coastal upwelling areas and OMZs, canonical denitrification seems to be the main N_2_O removal mechanism, but it may be closely followed by assimilative N_2_O fixation.

For comparison, Table S3 also includes selected stations, where published data of N_2_ fixation [40] and dissimilative N_2_O reduction to N_2_ (canonical denitrification) obtained during KN182-9 and Galathea-3 cruises [39, 92, 93] are available. Notably, despite the different analytical techniques used, similar dissimilative N_2_O reduction rates were reported, fluctuating from 0.02 to 16 nmol L^-1^ d^-1^ [39], whereas N_2_ fixation ranged from 0.01 to 3.27 nmol L^-1^ d^-1^ [40].

References

89. Nightingale PD, Malin G, Law CS, Watson AJ, Liss PS, et al. (2000) In situ evaluation of air-sea gas exchange parameterizations using novel conservative and volatile tracers. Global Biogeochem Cy 14: 373-387. doi: 10.1029/1999gb900091. Available: <http://dx.doi.org/10.1029/1999GB900091>. Accessed 2013 April 23.

90. Wanninkhof R (1992) Relationship Between Wind Speed and Gas Exchange. J Geophys Res 97: 7373-7382.doi: 10.1029/92JC00188. Available: <ftp://ftp.etl.noaa.gov/users/cfairall/bulkalg/gasflux/papers/wanninkhof%20jgr%201992.pdf>. Accessed 2013 April 23.

91. Garratt JR (1977) Review of Drag Coefficients over Oceans and Continents. Mon Weather Rev 105: 915-929. doi: 10.1175/1520-0493(1977)105<0915:RODCOO>2.0.CO;2. Available: [http://dx.doi.org/10.1175/1520-0493(1977)105<0915:RODCOO>2.0.CO;2](http://dx.doi.org/10.1175/1520-0493(1977)105%3c0915:RODCOO%3e2.0.CO;2). Accessed 2013 April 23.

92. Chang BX, Devol AH, Emerson SR (2010) Denitrification and the nitrogen gas excess in the eastern tropical South Pacific oxygen deficient zone. Deep-Sea Res Pt I: Oceanographic Research Papers 57: 1092-1101. doi: 10.1016/j.dsr.2010.05.009. Available: <http://www.sciencedirect.com/science/article/pii/S0967063710001263>.Accessed 2013 April 23.

93. Dalsgaard T, Thamdrup B, Farías L, Revsbech NP (2012) Anammox and denitrification in the oxygen minimum zone of the eastern South Pacific. Limnol Oceanogr 57: 1331-1346. doi: 10.4319/lo.2012.57.5.1331. Available: <http://www.aslo.org/lo/toc/vol_57/issue_5/1331.html> .Accessed 2013 April 23.
